# MoMkt1, a member of XPG/RAD2 nuclease family, regulates development and pathogenicity in *Magnaporthe oryzae*

**DOI:** 10.1080/21505594.2025.2546068

**Published:** 2025-08-25

**Authors:** Na Li, Junlian Xiao, Long Yan, Xiaoru Kang, Wenjuan Wang, Shen Chen, Weihuai Wu, Shulin Zhang

**Affiliations:** a Anhui Province Key Laboratory of Crop Integrated Pest Management/College of Plant Protection, Anhui Agricultural University, Hefei, Peoples’ Republic of China; b Hainan Key Laboratory for Monitoring and Control of Tropical Agricultural Pests, Environment and Plant Protection Institute, Chinese Academy of Tropical Agricultural Sciences, Haikou, Peoples’ Republic of China; c Guangdong Provincial Key Laboratory of High Technology for Plant Protection, Plant Protection Research Institute, Guangdong Academy of Agricultural Sciences, Guangzhou, Peoples’ Republic of China

**Keywords:** *Magnaporthe oryzae*, XPG/RAD2 nuclease family, MoMkt1, DNA repair, pathogenicity

## Abstract

XPG/RAD2 nuclease family plays a crucial role in DNA damage repair to maintain genomic integrity. However, the biological function of Mkt1, a member of the XPG/RAD2 nuclease family, remains unclear in *Magnaporthe oryzae*. In this study, we identified and characterized the biological functions of MoMkt1. Our results demonstrated that MoMkt1 is involved in hyphal growth, conidiation, normal appressorium development, degradation of glycogen and lipid droplets, cell wall stress and oxidative stress responses, and pathogenicity in *M. oryzae*. Additionally, MoMkt1 is required for scavenging host-derived reactive oxygen species (ROS) and responding to DNA replicative stress. Further investigation revealed that MoMkt1 interacts with the transcription factor IIH (TFIIH) core subunit MoTfb2. Overexpression (OE) of *MoTFB2* slightly affected the virulence of *M. oryzae*. Transcriptome data revealed that *MoMKT1* regulates metabolic pathways, cell wall, oxidation-reduction processes, and DNA repair. Our study provides insights into MoMkt1-mediated development and pathogenicity in *M. oryzae*.

## Background

Genome stability is crucial for the survival of all organisms. DNA replication, chromosome segregation, and DNA repair are involved in maintenance of genome stability [1]. DNA damage, including double-stranded DNA breaks (DSBs), single-stranded DNA breaks (SSBs), chemical modifications, formation of apurinic/apyrimidinic (AP) sites, intra- and inter-strand crosslinks, and protein-DNA adducts, can impair genomic integrity and cell viability [2,3]. Organisms have evolved conserved signal transduction pathways to repair DNA damage, also called as DNA damage response (DDR). There are at least five repair pathways based on the type of DNA damage: base excision repair (BER), nucleotide excision repair (NER), mismatch repair (MMR), nonhomologous end joining (NHEJ), and homologous recombination (HR) [2].

Nucleases have been reported to play critical roles in replication, recombination and repair processes. XPG/RAD2 nuclease family was identified as the first eukaryotic nuclease family with unique nuclease activities that contribute to genome stability [4]. Based on nuclease activity and sequence homology, the XPG/RAD2 nuclease family is divided into five classes: xeroderma pigmentosum group G protein (*XPG*)/class I, flap structure-specific endonuclease 1(*FEN-1*)/class II, exonuclease 1(*EXO-1*)/class III, *Oryza sativa* single-strand DNA endonuclease 1 (*OsSEND-1*)/class IV, and *Drosophila melanogaster* GEN1 Holliday junction 5’flap endonuclease (*DmGEN*)/class V [5]. Class I XPG homologs cleave the 3´-side of damaged DNA and are involved in NER, BER, or homologous recombination repair (HRR) [6,7]. In humans, *XPG* deletion leads to the inherited disorder xeroderma pigmentosum, and some affected individuals exhibit Cockayne syndrome [8]. *RAD2*, a homolog of human *XPG*, promotes efficient RNA polymerase II transcription in *Saccharomyces cerevisiae* [9]. Class II consists of *FEN-1* homologs, which have the activities of 5´-exonuclease and gap endonuclease and contribute to multiple DNA metabolic pathways [4,10]. In humans, OE of *FEN1* completely rescues the miR-140-induced DNA repair defects [11]. In mice, *FEN-1* deletion shows embryonic lethality [12]. In *S. cerevisiae*, RAD27 deletion results in increased sensitivity to UV light and the alkylating agent methyl methane sulfonate (MMS) [13]. Class III is the *EXO-1* homologs which incises DNA in the 5´-3´ direction extensively and participates in various metabolic processes, such as DNA recombination, MMR, NER, and telomere maintenance [14]. In mice, *EXO1* deletion increases susceptibility to cancer and sterility [15]. OsSEND-1 has a single-stranded endonuclease, whereas DmGEN has endo-exonuclease activities on single-stranded DNA, double-stranded DNA, gapped double-stranded DNA and nicked double-stranded DNA [16]. In Rice, the transcriptional level of *OsSEND-1* is induced by UV and DNA-damaging agents, such as Methyl Methane sulfonate (MMS), or H_2_O_2_ [17]. In addition, the multi-subunit transcription factor IIH (TFIIH) complex is responsible for NER and transcription initiation via RNA polymerase II transcription [18]. TFIIH consists of two subcomplexes: the 7-subunit core TFIIH subcomplex (Ssl2/XPB, Rad3/XPD, Tfb1/p62, Tfb2/p52, Ssl1/p44, Tfb4/p34, and Tfb5/p8) and the 3-subunit CDK-activating kinase (Tfb3/Mat1, Kin28/Cdk7, and CycH/CcnH) [19,20]. Previous studies have reported that TFIIH recruits the 3´-endonuclease XPG/Rad2 to participate in DNA repair in the NER pathway [21]. These studies suggest that the XPG/RAD2 nuclease family plays an important role in eukaryotic DNA repair.

Mkt1 is a member of the XPG/RAD2 endonuclease family of proteins that contain an N-terminal XPG-like domain (PIN domain) [22]. A substantial body of literature has documented the biological function of *MKT1* in various organisms. In *Trypanosoma brucei*, *MKT1* plays an important role in mRNA metabolism and gene expression by interacting with RNA-binding proteins [23,24]. In *S. cerevisiae*, *MKT1* is required for replication or maintenance of M_2_ double-stranded RNA at temperatures above 30°C, and regulates *HO* expression at the post-transcriptional level [22,25]. In addition, hybrid progeny from a BY × RM cross has different sensitivities to the DNA damage agent 4-nitroquinoline 1-oxide (4-NQO), Demogines et al. [26] showed that *MKT1* is associated with 4-NQO resistance [26]. *MKT1* is involved in RNAi-mediated post-transcriptional silencing of *Schizosaccharomyces pombe* [27]. In *Cryptococcus neoformans*, *MKT1* is involved in sexual reproduction and virulence. *MKT1* deletion mutants impede hyphal elongation during sexual reproduction and virulence in mice [28]. Rice blast, caused by *Magnaporthe oryzae*, is one of the most important diseases that threatens crop production and is responsible for global economic loss [29]. However, the biological functions and regulatory mechanisms of Mkt1 in *M. oryzae* remain unclear.

In this study, the biological functions of MoMkt1 were identified and characterized. Functional analysis indicated that MoMkt1 is involved in hyphal growth, conidiation, appressorium formation, degradation of glycogen and lipid droplets, responses to cell wall stress and oxidative stress as well as pathogenicity in *M. oryzae*. Additionally, MoMkt1 is required for scavenging host-derived ROS and responding to DNA replicative stress. It was further found that MoMkt1 interacts with the TFIIH core subunit MoTfb2. *MoTFB2*-overexpressed slightly affected the virulence of *M. oryzae*. Transcriptome data revealed that *MoMKT1* regulates metabolic pathways, cell wall, oxidation-reduction processes, and DNA repair. Our study provides insights into the MoMkt1-mediated development and pathogenicity of *M. oryzae*.

## Materials and methods

### Strains, culture conditions and phenotypic analysis

The wild-type (WT) strain Guy11 and all strains generated in this study were cultured on complete medium (CM) medium at 28°C. *Escherichia coli* strain DH5α and *Agrobacterium tumefaciens* C58 strain AGL1 were used for bacterial transformation. Vegetative growth, conidiation, appressorium formation, pathogenicity, and penetration assays were performed according to a previously reported method [30].

### Plasmid constructs and fungal transformants

To obtain the ∆*Momkt1* mutant, a *MoMKT1* knockout vector was constructed according to a previously reported method [31]. Briefly, about 1200 bp upstream fragment and downstream fragment of *MoMKT1* were amplified from the WT Guy11 genomic DNA. The upstream fragment, downstream fragment, and the hygromycin resistance cassette (*HPH*) fragment derived from the pFGL821 plasmid [32] were ligated into the *Hin*dIII/*Xba*I-linearized vector pKO1B [31] using a one-step cloning kit (Vazyme, China). The correct vector was transformed into the WT strain Guy11 using *Agrobacterium tumefaciens* C58 strain AGL1(Weidi, China) via *Agrobacterium tumefaciens*-mediated transformation (AtMT). Putative *MoMKT1* deletion mutants were screened by polymerase chain reaction (PCR), reverse transcription polymerase chain reaction (RT-PCR) and Southern blotting. To generate the complemented strain *∆Momkt1/MoMKT1*, the DNA sequence containing full-length *MoMKT1* without a stop codon and its native promoter was cloned into *Xho*I-linearized pYF11 (bleomycin resistance) to produce the MoMkt1-GFP expression vector and transformed into the *∆Momkt1* mutant, as described in previous studies [33,34]. To construct the *MoTFB2* OE strain, full-length *MoTFB2* without a stop codon was amplified from the WT strain Guy11 genomic DNA using primer pairs MoTfb2_pKD3_F/R, and cloned into the *BamH*I/*Sma*I-linearized pKD3-GFP vector [35] using the ClonExpress II One Step Cloning Kit (Vazyme, China). The resulting vector was transformed into the WT strain Guy11 using AtMT, and the transformants were screened for bialaphos resistance and further verified by RT-PCR using specific primers MoTfb2_RT_F/R. All the primers used are listed in Table S1.

### RNA isolation, RT-PCR and qRT-PCR

Total RNA of all tested strains was isolated from mycelia and extracted using TRIzol reagent (Invitrogen, USA), according to the manufacturer’s protocols. cDNA synthesis was performed using the HiScript II 1st Strand cDNA Synthesis Kit (+gDNA wiper) (Vazyme, China). RT-PCR was performed to confirm the Δ*Momkt1* mutants, the complemented strain Δ*Momkt1/MoMKT1*, and OE-*MoTFB2* using the gene-specific primers MoMkt1_RT_F/R and MoTfb2_RT_F/R, respectively. Quantitative real-time PCR (qRT-PCR) was performed as previously described [36]. The *β‐tubulin* gene *(MGG_00604)* was used as an endogenous reference. The primers used are listed in Table S1.

### Subcellular localization analysis of MoMkt1

For subcellular localization of MoMkt1 in *M. oryzae*, the MoMkt1-GFP expression vector was transformed into the WT strain Guy11, and GFP signals were observed at different developmental stages of *M. oryzae* using a laser scanning confocal microscope (Nikon, Japan). To further verify the subcellular localization of MoMkt1, a nuclear marker vector H1-RFP was introduced into the WT strain Guy11 along with MoMkt1-GFP. GFP and RFP signals were observed using a laser scanning confocal microscope (Nikon, Japan).

### Staining assays

ROS and diphenyleneiodonium (DPI) staining assays were performed with 3, 3´‐diaminobenzidine (DAB) solution and 0.5 μM DPI, respectively [30]. For lipid droplet or glycogen staining, Nile Red solution or KI/I_2_ solution was used to stain conidia, germ tubes and appressoria after induction on a hydrophobic surface for 0, 2, 8, 16, and 24 hours, and the samples were observed under a fluorescence microscope, respectively.

### Stress assays

For stress assays, mycelial blocks of all tested strains were cultured on CM plates supplemented with 200 µg/mL calcofluor white (CFW), 600 µg/mL Congo red (CR), 0.01% sodium dodecyl sulfate (SDS), 1 M sorbitol, 0.7 M NaCl, 0.6 M KCl, 5 mM H_2_O_2_, 10 mM H_2_O_2_, and 10 mM hydroxyurea (HU) at 28°C for 7 days. Subsequently, the colonies were photographed, and their diameters were measured after 7 days. The relative inhibition rate was calculated using the following formula: Relative inhibition rate = (diameter of the untreated strain – diameter of the strain treated with chemicals)/(diameter of the untreated strain).

### Bimolecular fluorescence complementation (BiFC) assay

A full-length *MoMKT1* without a stop codon was cloned into *Xba*I-linearized pKD2-YFP^NTF^ (hygromycin B resistance), and the open reading frame (ORF) of *MoTFB2* without a stop codon was cloned into *BamH*I-linearized pkD5-YFP^CTF^ (chlorimuron-ethyl resistance) using a one-step cloning kit (Vazyme, China) as previously described [37]. MoMkt1-YFP^NTF^ and YFP^CTF^-MoTfb2 were co-transferred into the WT strain Guy11 using the AtMT method. Fluorescence signals were observed in mycelia under a laser scanning confocal microscope (Nikon, Japan).

### Co-immunoprecipitation (Co-IP) assays

A full-length *MoTFB2* sequence lacking a stop codon was amplified and cloned into a *Hin*dIII-linearized pKNRP27Flag vector (which contains a 3×Flag tag and confers neomycin resistance) using a one-step cloning kit (Vazyme, China) as described previously [38]. Subsequently, the MoTfb2–3×FLAG was introduced into the WT strain Guy11, which expressed MoMkt1-GFP, using the polyethylene glycol (PEG)-mediated protoplast transformation method. Total protein was extracted from mycelia of co-expressing MoMkt1-GFP and MoTfb2‐3×FLAG strain and then incubated with GFP beads (AlpalifeBio, China) for 3 hours at 4°C. Bound proteins were eluted from the GFP-Trap beads for western blot analysis. The eluted protein samples were detected using anti-GFP or anti-FLAG antibodies (Proteintech, China). A pre-stained protein ladder (Biomed, China) was used as the molecular marker for protein identification.

### Transcriptome analysis

Mycelia of the WT strain Guy11 and the Δ*Momkt1* mutant with three biological replicates were harvested after culturing in liquid CM medium with shaking at 150 rpm for 48 hours at 28°C and then sent to GENE DENOVO (Guangdong, China) for total RNA extraction, mRNA purification, cDNA synthesis, and RNA sequencing. The DESeq2 package was used for the differentially expressed genes (DEGs) analysis. DEGs were filtered using a threshold of log_2_(fold change [FC]) > 1 and *p*_adj_ < 0.05, a false discovery rate (FDR) [39]. For gene functional annotation, Gene Ontology (GO) enrichment analysis and Kyoto Encyclopedia of Genes and Genomes (KEGG) pathway analysis of DEGs were performed using the topGO R and clusterProfiler R package [40,41].

### Statistical analysis

Mean values ± standard deviation (SD) were calculated from three independent biological replicates, each with a minimum of three technical replicates. Statistical significance was assessed using a two-sample Student’s t-test conducted using Microsoft Office Excel software.

## Results

### Identification and subcellular localization of MoMkt1 in M. oryzae

**Figure 1.**
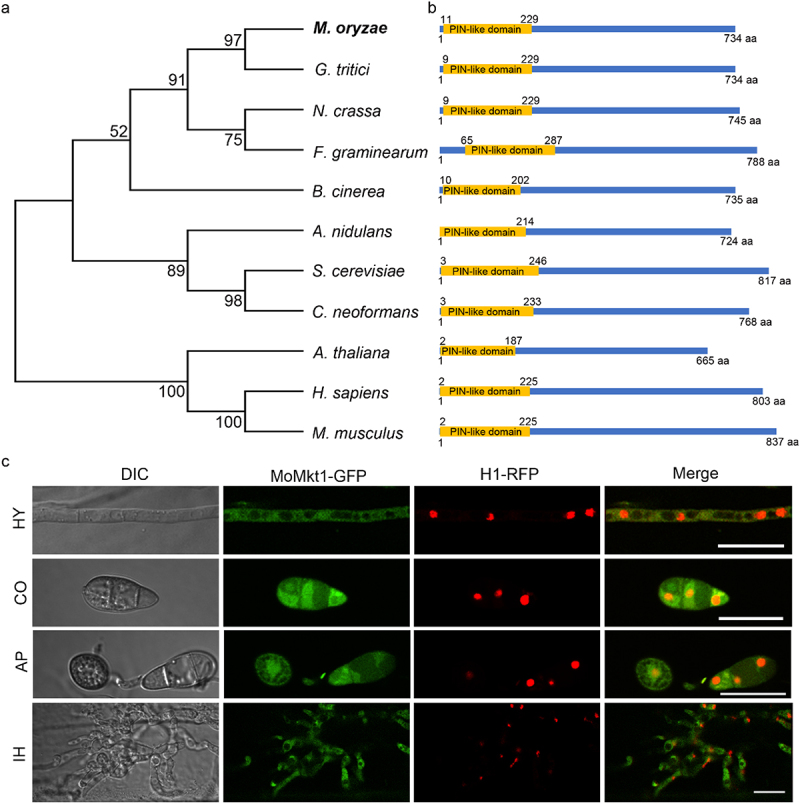
Characterization and subcellular localization of MoMkt1 protein in *M. oryzae*. (a) Phylogenetic analysis of MoMkt1 and its homologs from different organisms using the neighbor-joining method by MEGA software. GenBank accession numbers and the corresponding species names are as follows: XP_003715774.1 (*Magnaporthe oryzae*); XP_009223415.1 (*Gaeumannomyces tritici*); XP_960742.1 (*Neurospora crassa*); KAI6766631 (*Fusarium graminearum*); XP_024550529.1 (*Botrytis cinerea*); XP_050469344.1 (*Aspergillus nidulans*); XP_567528.1 (*Cryptococcus neoformans*); NP_174256.2 (*Arabidopsis thaliana*); KAI4085534.1 (*Homo sapiens*); NP_036142.2 (*Mus musculus*); NP_014314.3 (*Saccharomyces cerevisiae*). (b) Protein domain analysis of Mkt1 was performed using pfam website (https://www.ebi.ac.uk/interpro/). The amino acid (aa) residue number at the start and end of the protein, as well as the predicted domains, were labeled at the top or bottom of the protein. (c) Subcellular localization distribution of MoMkt1 at different developmental stages. Transformant expressing MoMkt1-GFP and H1-RFP plasmids in the WT strain Guy11 was observed using a laser scanning confocal microscope (Nikon, Japan). HY, hypha; CO, Conidium; AP, appressorium at 6 hpi; IH, invasive hyphae at 36 hpi. Bar, 20 μm.

To obtain the homolog of the Mkt1 in *M. oryzae*, we used *S. cerevisiae* Mkt1 amino acid sequence as a query to perform a BLASTp search of the *M. oryzae* genome database (http://fungi.ensembl.org/Magnaporthe_oryzae/Info/Index), and identified Mkt1 homologous protein (*734-amino acids*) encoded by *MGG_08303*. Therefore, *MGG_08303* was designated as *MoMKT1* in *M. oryzae*. Phylogenetic analysis revealed that Mkt1 is conserved across various species and exhibits a close evolutionary relationship with Mkt1 in *Gaeumannomyces tritici* (Figure 1a). Domain prediction analysis revealed that MoMkt1 and its homolog contain a PIN-like domain (XPG domain) (Figure 1b). These results indicate that Mkt1 is conserved across eukaryotes.

To understand the subcellular localization of MoMkt1 at different developmental stages in *M. oryzae*, the MoMkt1-GFP fusion protein construct was transformed into the WT strain Guy11, and GFP signals were observed at different stages, including mycelia, conidia, appressoria (6 hours post-incubation [hpi]), and invasive hypha (36 hpi), using confocal microscopy. MoMkt1-GFP was localized in the cytoplasm at all tested stages (Figure 1c). To further confirm that MoMkt1-GFP was localized to the cytoplasm, the nuclear marker H1-RFP was co-expressed with MoMkt1-GFP in the WT strain Guy11. The fluorescence signals of H1-RFP did not colocalize with those of MoMkt1-GFP in any of the tested stages (Figure 1c). These results suggest that MoMkt1 is a cytoplasm-localized protein involved in the growth and development of *M. oryzae*.

### MoMKT1 is involved in vegetative growth and conidiation of M. oryzae

To explore the role of *MoMKT1* in *M. oryzae*, the *MoMKT1* deletion mutants were generated in the WT strain Guy11 with homologous recombination strategy (Figure S1a). Candidate *MoMKT1 deletion* mutant transformants were verified using PCR, RT-PCR, and Southern blotting (Figure S1b-d). We successfully obtained the *MoMKT1* deletion mutants (12, 14, 18, 19, 30, 34), and named them as ∆*Momkt1*. In addition, the complemented strain was constructed for the ∆*Momkt1* mutant by introducing *MoMKT1* with its native promoter into the Δ*Momkt1* mutant (19). The putative complementary strains were verified by RT-PCR analysis, and named as Δ*Momkt1/MoMKT1* (Figure S1c). The WT strain Guy11, ∆*Momkt1* mutants (19 and 30), and the complemented strain Δ*Momkt1/MoMKT1* were used for phenotypic analysis.

**Figure 2.**
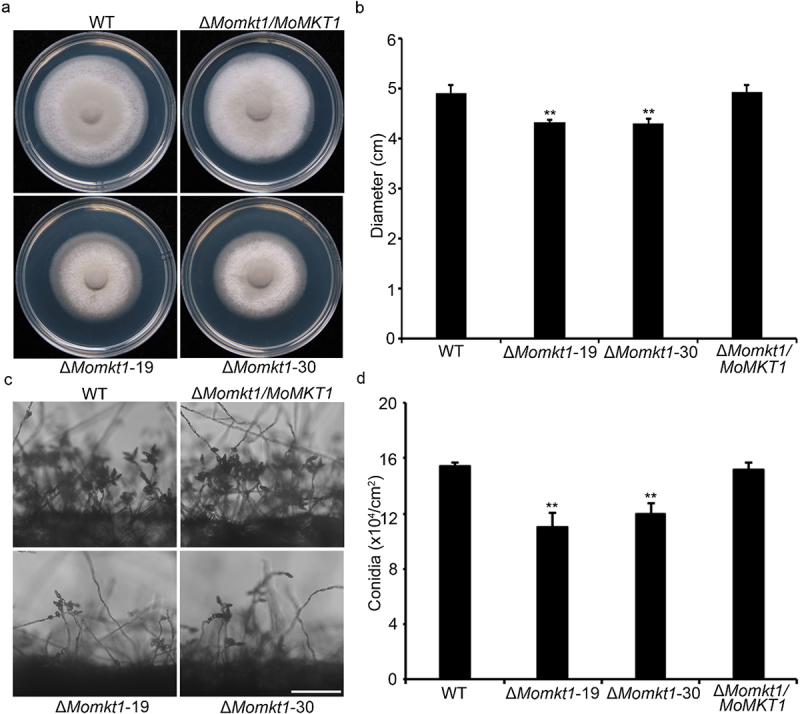
*MoMKT1* is involved in the vegetative growth and conidiation of *M. oryzae*. (a) Colonies of the WT strain Guy11, Δ*Momkt1* mutants, and the complemented strain Δ*Momkt1/MoMKT1* on CM plates were observed after 7 days at 28°C. (b) Statistical analysis of the colony diameter of the indicated strains. (c) Conidiophores of the indicated strains. Bar, 100 µm. (d) Statistical analysis of conidiation of the indicated strains. Error bars represent SD and asterisks indicate significant differences between the WT strain Guy11 and Δ*Momkt1* mutants, estimated using the Student’s *t*-test (**, *p* < 0.01).

To investigate the function of *MoMKT1* in *M. oryzae* fungal development, vegetative growth and conidiation analyses of the ∆*Momkt1* mutants were performed. The WT strain Guy11, the ∆*Momkt1* mutants, and the complemented strain Δ*Momkt1/MoMKT1* were cultured on CM plate for 7 days at 28°C under dark conditions. The ∆*Momkt1* mutants showed a significant reduction in colony diameter compared with the WT strain Guy11 and the complemented strain Δ*Momkt1/MoMKT1* (Figure 2a, b). We observed that the Δ*Momkt1* mutants produced fewer conidia on the conidiophores than the WT strain Guy11 and the complemented strain Δ*Momkt1/MoMKT1* (Figure 2c), and further found that conidiation of the Δ*Momkt1* mutants was significantly decreased (Figure 2d). Taken together, these results suggest that *MoMKT1* is required for vegetative growth and conidiation of *M. oryzae*.

### MoMKT1 is required for full virulence of M. oryzae

**Figure 3.**
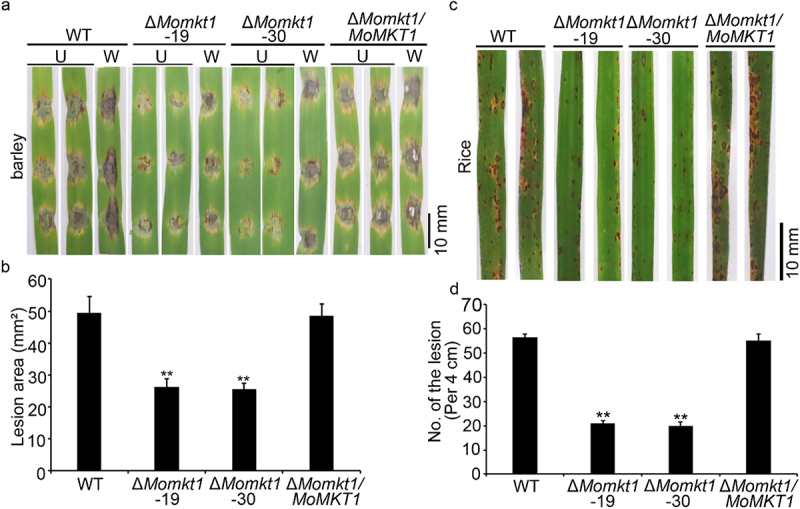
*MoMKT1* is important for full virulence of *M. oryzae*. (a) Pathogenicity was tested on unwounded (U) and wounded (W) barley leaves. The mycelial agar plugs of the indicated strains were inoculated on 7-day-old barley leaves and photographed at 5 dpi. Bar, 10 mm. (b) Statistical analysis of the lesion area of the indicated strains on barley leaves using the ImageJ software. Error bars represent SD and asterisks indicate significant differences (**, *p* < 0.01). (c) 14-day-old rice seedlings were inoculated by spraying with 10 mL conidial suspensions (5 × 10^4^ conidia/mL in a 0.2% (w/v) gelatin solution) from the indicated strains, and photographed at 5 dpi. Bar, 10 mm. (d) Statistical analysis of the number of lesions in the indicated strains within a 4 cm long leaf, and at least three leaves were counted for each strain. Three experiments were conducted. Error bars represent SD and asterisks indicate significant differences (**, *p* < 0.01).

To investigate the contribution of *MoMKT1* to the pathogenicity of *M. oryzae*, pathogenicity assays were performed on the susceptible barley *cv* Golden Promise and susceptible rice seedlings (*Oryza sativa cv* CO39). We first examined the virulence of all tested strains by inoculating detached barley leaves with mycelial plugs. At 5 days post-inoculation (dpi), mycelial plugs of the WT strain Guy11 and the complemented strain Δ*Momkt1/MoMKT1* caused severe lesions. However, the mycelial plugs of the *∆Momkt1* mutants had relatively small lesion areas. Even when inoculated into wounded barley, the *∆Momkt1* mutants resulted in a smaller lesion area (Figure 3a,b). To confirm the results of pathogenicity assays on barley with mycelial plugs, two-week-old rice seedlings were sprayed with conidial suspensions (5 × 10^4^ conidia/mL) of the WT strain Guy11, the *∆Momkt1* mutants, and the complemented strain *∆Momkt1/MoMKT1*. After 5 dpi, the lesions produced by the *∆Momkt1* mutants were smaller and fewer than those produced by the WT strain Guy11 and the complemented strain *∆Momkt1/MoMKT1* (Figure 3c,d). These results demonstrated that *MoMKT1* is required for the full pathogenicity of *M*. *oryzae*.

### MoMKT1 functions in appressorium formation, invasive hyphal expansion, and scavenging host-derived ROS

**Figure 4.**
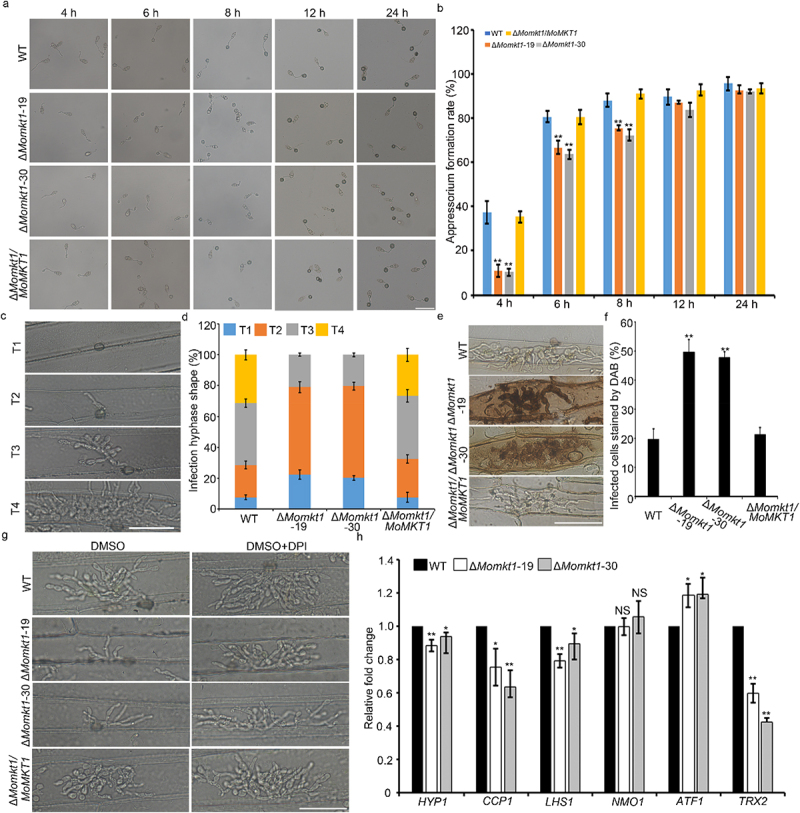
*MoMKT1* is important for appressorium formation, invasive hyphal expansion, and scavenging host ROS. (a) Appressorium formation in the WT strain Guy11, Δ*Momkt1* mutants, and the complemented strain Δ*Momkt1/MoMKT1* was induced on an artificial hydrophobic surface, and observed at 4, 6, 8, 12, and 24 hpi. Bar, 50 µm. (b) Statistical analysis of the appressorium formation rates of the tested strains. A total of 100 conidia were counted for each strain and three experiments were performed. (c) Four types of IH of *M. oryzae* in barley epidermal cells. Type 1 (T1), no penetration; type 2 (T2), single infectious hypha; type 3 (T3), more than two IH limited to one cell; type 4 (T4). IH extended to the adjacent cells. Bar, 50 μm. (d) Statistical analysis of each type IH of the indicated strains. A total of 100 infectious penetration sites were counted for each strain, and the experiment was repeated thrice. (e) DAB staining was performed on the infected barley leaves of the indicated strains at 30 hpi. Bar, 50 μm. (f) Statistical analysis of DAB-stained infected barley cells. For each strain, at least 100 infected barley cells were observed and three experiments were performed. (g) Barley leaves were inoculated with conidial suspensions of all tested strains treated with DPI, and invasive hyphae were observed at 30 hpi. The dimethyl sulfoxide (DMSO) was used as a control to dissolve the DPI. Bar, 50 μm. (h) Transcription level of ROS-detoxification‐related genes in the indicated strains. The *β‐tubulin* gene (*MGG_00604*) was used as the reference gene. Three independent biological experiments were conducted. Error bars represent SD and asterisks indicate significant differences (**, *p* < 0.01; *, *p* < 0.05). “NS” represents non-significant differences (NS, *p* > 0.05).

To analyze how *MoMKT1* affects the virulence of *M. oryzae*, we first counted the appressorium formation rates of the WT strain Guy11, the Δ*Momkt1* mutants, and the complemented strain Δ*Momkt1/MoMKT1* at different stages (4, 6, 8, 12, and 24 hours). The appressorium formation rate of the Δ*Momkt1* was remarkably lower than that of the WT strain Guy11 and the complemented strain *∆Momkt1/MoMKT1* at 4, 6, and 8 hpi. However, the appressorium formation rates of the Δ*Momkt1* mutants, the WT strain Guy11, and the complemented strain *∆Momkt1/MoMKT1* became indistinguishable at 12 and 24 hpi (Figure 4a,b). These results demonstrated that *MoMKT1* is important for appressorium development.

To further investigate the cause of the pathogenicity defect in the Δ*Momkt1* mutants, a penetration assay was performed on detached barley leaves. Approximately 28% of the WT strain Guy11 and the complemented strain *∆Momkt1/MoMKT1* exhibited invasive hyphal (IH) spread to neighboring barley cells at 36 hpi. However, IH from the Δ*Momkt1* mutants were mainly limited to the initially infected cells (Figure 4c,d). These results indicated that *MoMKT1* plays a role in the expansion of infectious hyphae. The results presented above indicate that the hyphal infectious expansion of the Δ*Momkt1* mutants was restricted to barley cells. Host-derived ROS generation can prevent pathogen invasion [42]. We hypothesized that the ability to scavenge host-derived ROS would be impaired in the Δ*Momkt1* mutants. Therefore, we conducted a DAB staining assay to detect ROS accumulation at the infection site. The results of this assay indicated that 50% of barley cells infected with the Δ*Momkt1* mutants were stained, whereas 20% and 21% of barley cells infected with the WT strain Guy11 and the complemented strain *∆Momkt1/MoMKT1*, respectively (Figure 4e,f). Furthermore, we used the NADPH oxidase inhibitor DPI in barley epidermal cells, and found that the expansion capability of the Δ*Momkt1* mutants IH was comparable with that of the WT strain Guy11 and the complemented strain *∆Momkt1/MoMKT1* (Figure 4g). In addition, the expression levels of several ROS detoxification-related genes were detected, including *ATF1*, *HYR1*, *TRX2*, *CCP1*, *NMO1*, and *LHS1* [43–46], in the WT strain Guy11 and the Δ*Momkt1* mutants. We found that the expression levels of *HYP1, CCP1, LHS1*, and *TRX2* were significantly downregulated in the Δ*Momkt1* mutants compared with those in the WT strain Guy11. The expression level of *ATF1* was upregulated (Figure 4h). Taken together, our results indicated that *MoMKT1* plays a role in scavenging ROS from the host.

### Disruption of MoMKT1 delays the mobilization and degradation of glycogen and lipid droplets

**Figure 5.**
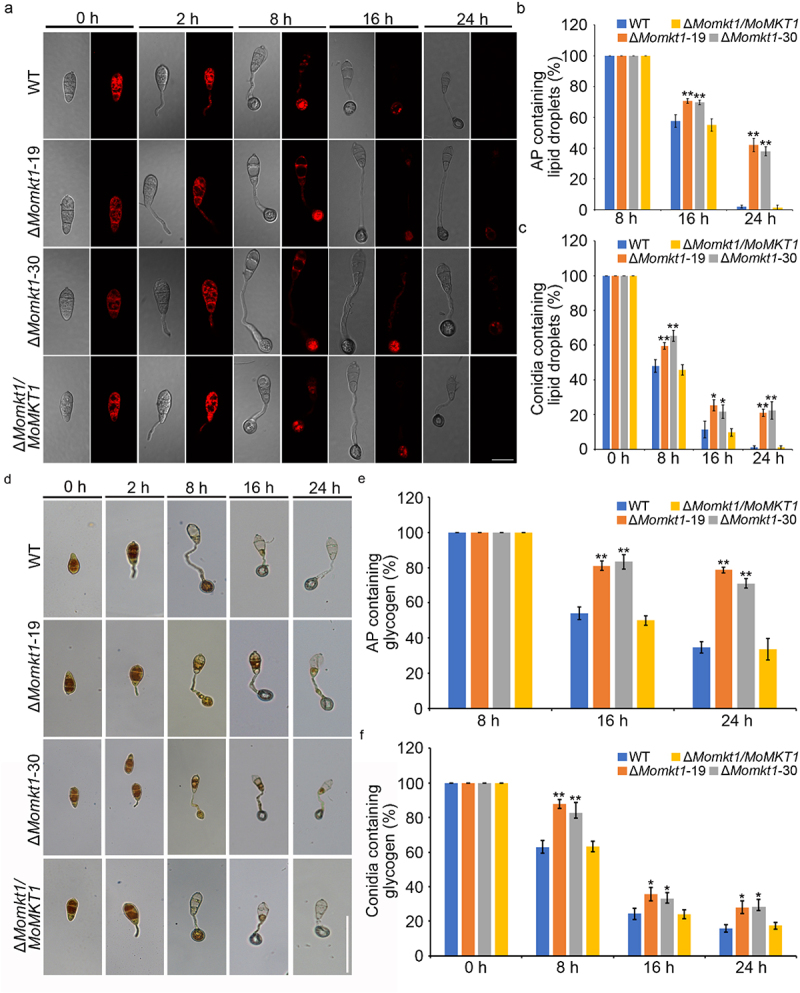
Disruption of MoMkt1 delays the mobilization and degradation of glycogen and lipid droplets. (a) Distribution of lipid droplets in conidia and appressorium of all tested strains stained with Nile red solution was observed under a light microscope at 0, 2, 8, 16 and 24 hours, respectively. Bar, 20 µm. (b) Statistical analysis of appressorium-containing lipid droplets in all tested strains. In total, 100 appressoria were counted. (c) Statistical analysis of conidia containing lipid droplets in all tested strains. A total of 100 conidia were counted in each experiment. (d) Distribution of glycogen in the conidia and appressorium of all tested strains stained with KI/I_2_ solution was observed under a light microscope at 0, 2, 8, 16, and 24 hours, respectively. Bar, 50 µm. (e) Statistical analysis of appressorium-containing glycogen in all the tested strains. In total, 100 appressoria were counted. Three independent biological experiments were conducted. (f) Statistical analysis of glycogen-containing conidia in all the tested strains. A total of 100 conidia were counted for each experiment. Error bars represent SD and asterisks indicate significant differences (**, *p* < 0.01; *, *p* < 0.05).

Glycogens and lipids stored in the conidia are critical for appressorium maturation [47]. Therefore, the movement and utilization of glycogen and lipids during appressorium maturation were observed by staining with KI/I_2_ and Nile red solutions, respectively. As shown in Figure 5a, the lipid distribution was similar in the conidia and germ tubes of all tested strains induced on a hydrophobic surface for 0–2 hours. At 8 hours, lipids in conidia were transferred to the appressorium in the WT strain Guy11 and the complemented strain *∆Momkt1/MoMKT1*. At 16–24 hours, lipids in the conidia and appressorium were rapidly consumed in the WT strain Guy11 and *∆Momkt1/MoMKT1*. In contrast, even at 24 hours, the conidia and appressorium of the Δ*Momkt1* mutants still retained lipids (Figure 5a-c). The dynamic distribution pattern of glycogen was similar to that of lipids in all the tested strains (Figure 5d-f). These results indicated that *MoMKT1* is essential for glycogen and lipid metabolism in *M. oryzae*.

### MoMKT1 is involved in different stress responses

**Figure 6.**
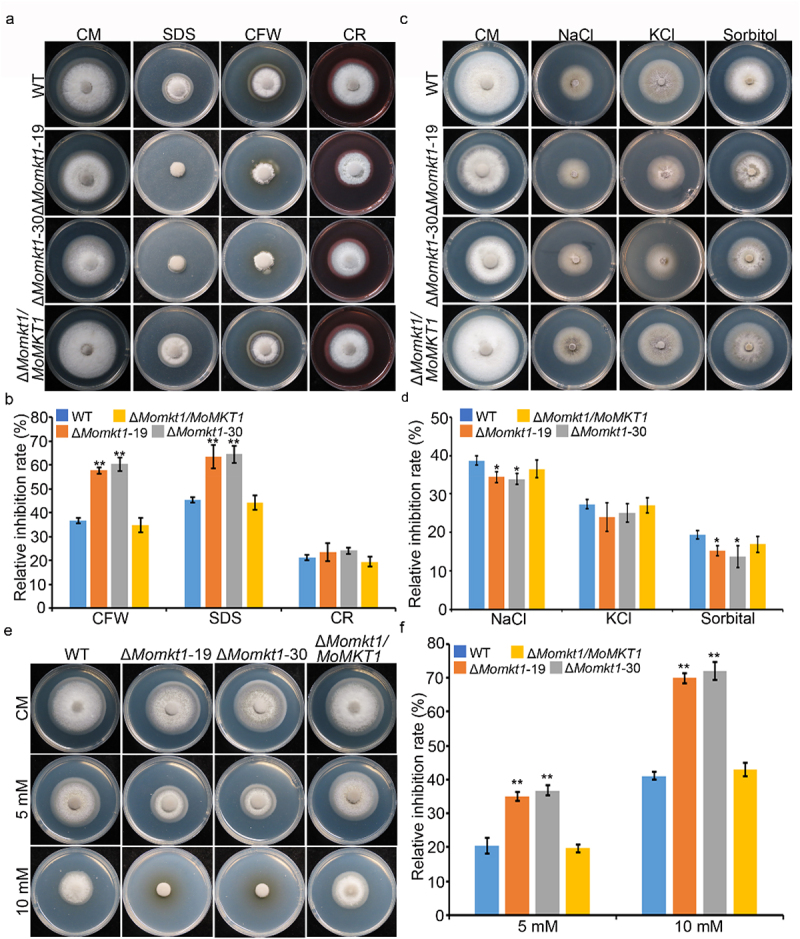
MoMkt1 is required for the cell wall stress response. (a) Colony morphology of the WT strain Guy11, Δ*Momkt1* mutants, and the complemented strain *∆Momkt1/MoMKT1* were cultured on CM containing 200 µg/mL CFW, 0.01% SDS, and 600 µg/mL CR. The colonies were measured and photographed under dark conditions at 28°C for 7 days. (b) Statistical analysis of the relative inhibition rates (%) of the indicated strains. (c) Colony morphology of the tested strains on CM containing 0.7 M NaCl, 0.6 M KCl, and 1 M sorbitol in the dark at 28°C for 7 days. (d) Statistical analysis of the relative inhibition rates (%) of the indicated strains. (e) Colony morphology of the tested strains on CM containing 5 mM H_2_O_2_ and 10 mM H_2_O_2_ in the dark at 28°C for 7 days. (f) Statistical analysis of the relative inhibition rates (%) of the indicated strains. For each strain, three independent biological experiments were performed, with three replicates each time. Error bars represent SD and asterisks indicate significant differences (**, *p* < 0.01; * *p* < 0.05). “NS” represents non-significant differences (NS, *p* > 0.05).

To investigate the role of *MoMKT1* in the stress response, the mycelial growth of the WT strain Guy11, the Δ*Momkt1* mutant, and the complemented strain *∆Momkt1/MoMKT1* was observed on CM supplemented with cell wall stress agents (600 µg/mL CR, 200 µg/mL CFW, and 0.01% SDS), osmotic stress agents (0.7 M NaCl, 1 M sorbitol, and 0.6 M KCl), and oxidative stress agents (5 mM and 10 mM H_2_O_2_). After 7 dpi, the colony diameters of all the tested strains were measured. The Δ*Momkt1* mutants were found to be sensitive to CFW and SDS, but more tolerant to NaCl, sorbitol, and KCl, and sensitive to both 5 mM and 10 mM H_2_O_2_ concentrations than that of the WT strain Guy11 and the complemented strain *∆Momkt1/MoMKT1* (Figure 6a-f). These results suggest that *MoMKT1* is involved in the cell wall stress and oxidative stress responses.

### MoMKT1 is required for the response to DNA replicative stress

**Figure 7.**
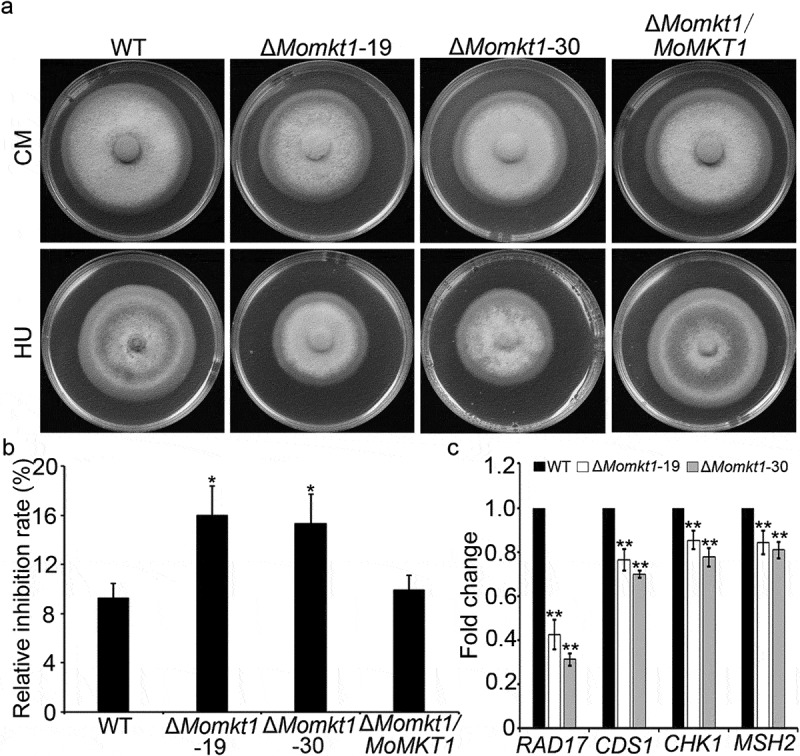
MoMkt1 is involved in DNA replicative stress response. (a) Colony morphology of the tested strains on CM containing 10 mM HU in the dark at 28°C for 7 days. (b) Statistical analysis of the relative inhibition rates (%) of the indicated strains. (c) Transcription of DNA repair-related genes in the indicated strains. The *β‐tubulin* gene (*MGG_00604*) was used as the reference gene. Three independent biological experiments were performed. Error bars represent SD and asterisks indicate significant differences (**, *p* < 0.01).

As shown in Figure 1b, MoMkt1 contains an N-terminal PIN-like domain (XPG domain). XPG/Rad2 endonuclease protein family participates in DNA repair in the NER pathway [48]. To verify whether *MoMKT1* is involved in DNA damage repair, we tested the sensitivity of the Δ*Momkt1* mutants to the DNA replication stress agent HU. The Δ*Momkt1* mutants showed significantly higher sensitively than the WT strain Guy11 and the complemented strain *∆Momkt1/MoMKT1* in the presence of HU (Figure 7a,b). Furthermore, we examined the transcription levels of DNA repair-related genes, including *MoMSH2*, which encodes a DNA MMR factor, and DNA damage checkpoint genes (*MoRAD17*, *MoCDS1*, and *MoCHK1*). As shown in Figure 7c, compared with the WT strain Guy11, the expression levels of these genes were significantly downregulated in the Δ*Momkt1* mutants. These results suggest that *MoMKT1* is required for the transcriptional regulation of DNA repair-related genes and response to DNA replication stress.

### MoMkt1 interacts with MoTfb2 in vivo

**Figure 8.**
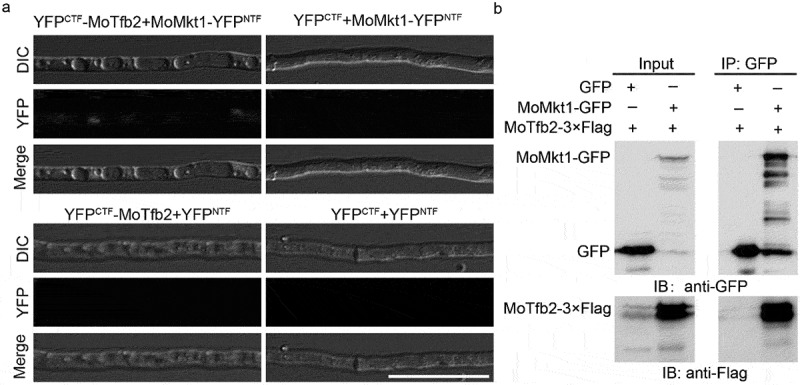
MoMkt1 interacts with MoTfb2 in *vivo*. (a) BiFC assay of the interaction between MoMkt1 and MoTfb2. Yellow fluorescent protein (YFP) signals were detected in vegetative hyphae expressing YFP^CTF^-MoTfb2 and MoMkt1-YFP^NTF^ using a laser scanning confocal microscope (Nikon, Japan). Strains expressing YFP^CTF^ and MoMkt1-YFP^NTF^, YFP^CTF^-MoTfb2 and YFP^NTF^, or YFP^CTF^ and YFP^NTF^ were used as negative controls. Bar, 20 µm. (b) Co-IP assay of the interaction between MoMkt1 and MoTfb2. MoMkt1-GFP and MoTfb2–3× FLAG were expressed in the WT strain Guy11. An empty GFP construct was used as a negative control. The Co-IP experiment was performed using anti-GFP beads, and the eluted proteins were analyzed by western blotting using anti-FLAG and anti-GFP antibodies.

Yeast Tfb2 (a core subunit of TFIIH) and its human ortholog, p52, are involved in both transcription and NER [49]. Therefore, we speculated that the XPG domain protein MoMkt1 interacts with the TFIIH core subunit Tfb2. To verify this hypothesis, we obtained the Tfb2 homolog in *M. oryzae* by using the *S. cerevisiae* Tfb2 amino acid sequence as a query to perform a BLASTp search of the *M. oryzae* genome database and identified *MoTFB2* (*MGG_00397*). The interaction between MoMkt1 and MoTfb2 was not detected in the yeast two-hybrid assay (Figure S2). We further performed BiFC and Co-IP assays and found that MoMkt1 interacts with MoTfb2 in vivo (Figure 8a,b).

**Figure 9.**
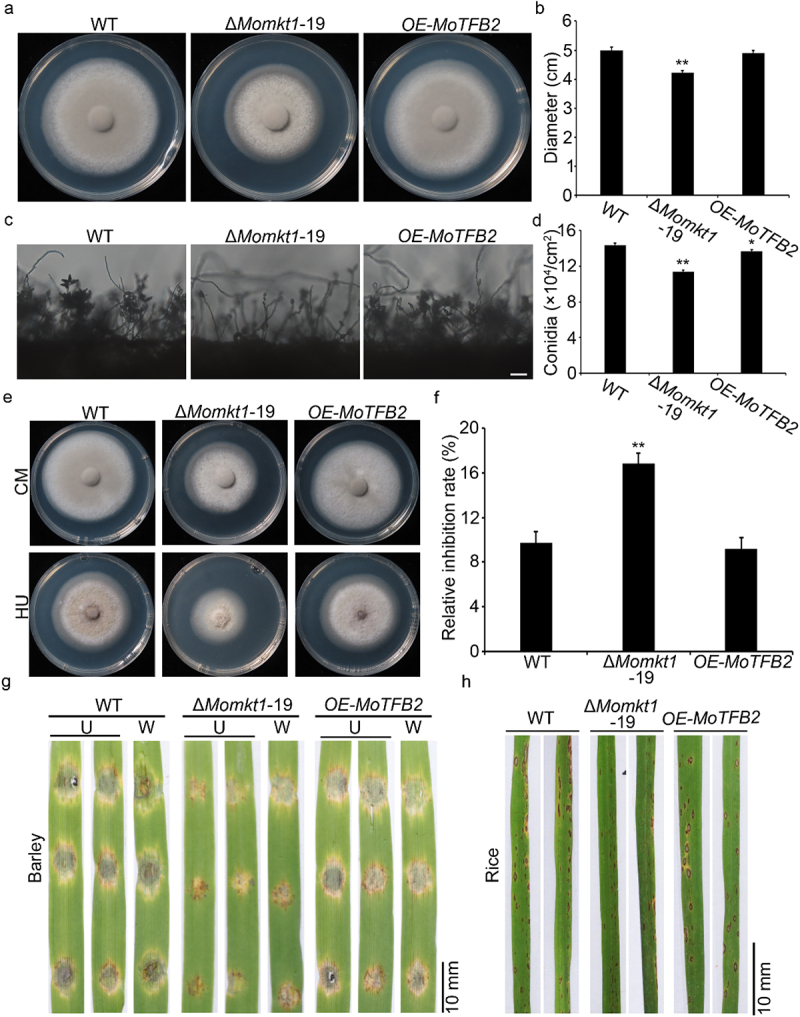
Phenotypic analysis of *MoTFB2* OE in *M. oryzae*. (a) Colony morphology of the WT strain Guy11, Δ*Momkt1* mutants, and OE strain *OE-MoTFB2* on CM plates was observed after 7 days at 28°C. (b) Statistical analysis of the colony diameters of the indicated strains. (c) Conidiophores of the indicated strains were observed under an inverted fluorescence microscope (Nikon, Japan). Bar, 20 µm. (d) Statistical analysis of conidiation of the indicated strains. (e) Colony morphology of the indicated strains on CM containing 10 mM HU at 28°C for 7 days. (f) Statistical analysis of the relative inhibition rates (%) of the indicated strains. For each strain, three independent biological experiments were performed, with three replicates. Error bars represent SD and asterisks indicate significant differences between the WT strain Guy11, Δ*Momkt1* mutant or *OE-MoTFB2* estimated using Student’s *t*-test (**, *p* < 0.01; *, *p* < 0.05). (g) Pathogenicity of the indicated strains was tested on unwounded (U) and wounded (W) barley leaves. Mycelial agar plugs were inoculated on 7-day-old barley leaves and photographed at 5 dpi. Bar, 10 mm. (h) 14-day-old rice seedlings were inoculated by spraying with 10 mL conidial suspensions (5 × 10^4^ conidia/mL in 0.2% (w/v) gelatin solution) from the indicated strains, and photographed at 5 dpi. Bar, 10 mm.

To further explore the function of *MoTFB2* in *M. oryzae*, we generated a targeted *MoTFB2* knockout vector *pKO1B::MoTFB2*, which was transformed into the WT strain Guy11. However, the Δ*Motfb2* mutant was unsuccessful, suggesting that *MoTFB2* may be an essential gene in *M. oryzae*. Therefore, we generated the *MoTFB2* OE strain *OE-MoTFB2* by expressing *MoTFB2* under the H3 promoter (Figure S3). Phenotypic analysis showed that *MoTFB2* OE did not affect vegetative growth or the response to HU (Figure 9a-f). However, statistical analysis of conidiation and pathogenicity tests showed that *OE-MoTFB2* caused fewer conidia and relatively smaller lesions than the WT strain Guy11 (Figure 9g-h). Taken together, these results revealed that MoMkt1 interacts with MoTfb2, regulating its pathogenicity in *M. oryzae*.

### MoMKT1 regulates various metabolic pathways in M. oryzae

**Figure 10.**
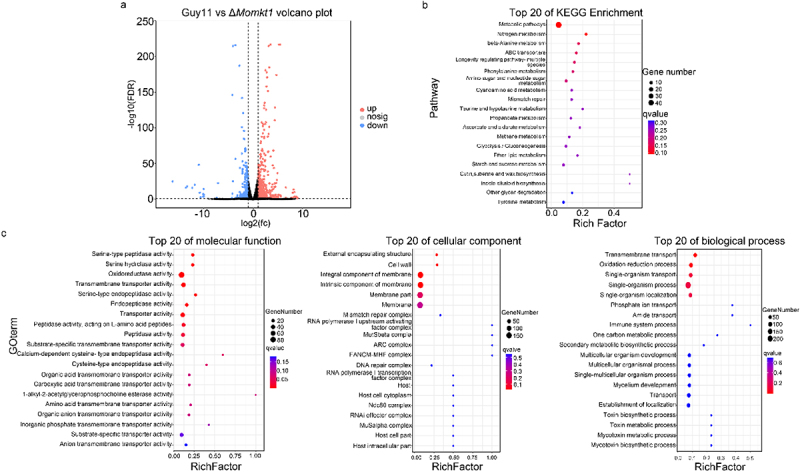
Transcriptome analysis of DEGs in comparison with the WT strain Guy11 and Δ*Momkt1* mutant. (a) DEGs in the WT strain Guy11 and Δ*Momkt1* mutant from RNA-seq data. ([FDR] < 0.05 and log_2_[FC] > 1). (b) Top 20 pathways from KEGG pathway enrichment analysis of significantly upregulated and downregulated genes. (c) Top 20 pathways from the GO pathway enrichment analysis of significantly upregulated and downregulated genes.

To further investigate the potential regulatory role of *MoMKT1* in *M. oryzae*, transcriptome analysis of the WT strain Guy11 and the Δ*Momkt1* mutant mycelia was performed using RNA sequencing (RNA-seq). RNA-seq analysis showed that compared with the WT strain Guy11, a total of 766 DEGs ([FDR] < 0.05, log_2_ [FC]) > 1), including 516 upregulated genes and 250 downregulated genes, were identified in the Δ*Momkt1* mutant (Figure 10a, Table S2). KEGG and GO enrichment analyses were performed to gain insight into the regulatory network of *MoMKT1*. KEGG pathway analysis of DEGs showed that the top 20 significantly enriched pathways included metabolic pathways, nitrogen metabolism, amino sugar and nucleotide sugar metabolism, ether lipid metabolism, and MMR (Figure 10b). GO enrichment analyses of DEGs indicated that oxidoreductase activity, transmembrane transporter activity, cell wall, MMR complex, DNA repair complex, transmembrane transport, and oxidation-reduction processes were significantly enriched (Figure 10c). To validate the authenticity of the RNA-seq results, 8 DEGs were randomly selected for qRT-PCR analysis, and the results were consistent with those of transcriptome analysis (Table S3). These results suggested that *MoMKT1* is involved in the regulation of metabolic pathways, cell wall, oxidation-reduction processes, and DNA repair.

## Discussion

XPG/RAD2 nuclease family plays an important role in DNA damage repair [4]. In mice, *XPG‐*deficiency affects left ventricular remodeling and dysfunction [50]. In rice, the expression of *OsEXO1*, which function in DNA repair, is induced by UV irradiation [51]. In *S. cerevisiae*, *YEN1* deletion increases the sensitivity of the Δ*mus81* mutants to DNA-damaging agents [52]. In this study, we characterized the XPG domain (PIN domain) protein MoMkt1 in *M. oryzae*. This domain is conserved among the XPG/RAD2 endonuclease family in eukaryotes, eubacteria, and archaea, and can bind RNA [22,53,54]. The PIN domains of E×O1and GEN1 interact with poly (ADP-ribose), which may participate in the DNA damage response [55]. Our results showed that MoMkt1 was localized in the cytoplasm and that *MoMKT1* deletion affected hyphal growth, conidiation, appressorium development, glycogen and lipid droplet degradation, responses to cell wall stress and DNA stress agents, and the fungal ability to penetrate the host in *M. oryzae*. Furthermore, we found that MoMkt1 interacted with MoTfb2, a core subunit of TFIIH. OE of *MoTFB2* affected the full virulence of *M. oryzae*. Transcriptome data revealed that *MoMKT1* regulates metabolic pathways, cell wall, oxidation-reduction processes, and DNA repair. This comprehensive study sheds light on the mechanism by which *MoMKT1* participates in DNA damage repair to regulate the development and virulence of *M. oryzae*.

DNA damage response is an effective way to deal with all types of DNA damage through repair mechanisms, including BER, NER, MMR, NHEJ, and HR [56]. As mentioned above, organisms contain many genes involved in DNA damage repair. In humans, mutations in BRCA1 and BRCA2 predispose individuals to breast and ovarian cancer [57]. In *Arabidopsis*, ARP5 participates in the repair of DNA damage treated with HU, MMS, and bleomycin, and *ARP5* deletion results in plant dwarfism [58]. In *S. cerevisiae*, the Δ*rpn4* mutant is sensitive to both proteotoxic and genotoxic agents [59]. In *M. oryzae*, DNA damage repair-related genes regulate the growth development and virulence. For example, MoKns1 is involved in regulating autophagy and DNA damage response pathways, thereby affecting virulence [60]. MoRfx1 regulates cell division during hyphal growth and conidial differentiation by mediating the expression of genes involved in DNA repair [61]. Mkt1 is a member of the XPG/RAD2 nuclease family and plays a role in genomic DNA metabolic processes [16]. Demogines et al. [26] showed that *MKT1* is associated with resistance to 4-NQO [26]. In *C. neoformans*, *MKT1* is involved in sexual reproduction and virulence [28]. In this study, *MoMKT1* deletion led to several phenotypic deficiencies. For example, the Δ*Momkt1* mutants exhibit a significant reduction in infection development and pathogenicity. In particular, the Δ*Momkt1* mutants showed a higher sensitivity to HU than the WT strain Guy11 and the complemented strain *∆Momkt1/MoMKT1*. We found that the transcription of DNA repair-related genes was significantly downregulated in the Δ*Momkt1* mutants. In conclusion, we provided evidence that *MoMKT1* is involved in the growth development and DNA repair of *M. oryzae*.

TFIIH contributes to many cellular processes, including NER and transcription initiation by RNA polymerase II [18,62]. During transcription, TFIIH functions in initiation, promoter escape, early elongation steps, and transcription re-initiation after RNA Pol II pausing [63]. During NER, TFIIH promotes DNA unwinding of an asymmetric region around the site of DNA lesions as an early step in excision and replacement of damaged DNA [64]. TFIIH has a flexible structure and a complex subunit composition. The Hub region of Tfb2 shows conserved cross-links with the Tfb4 and Ssl1 VWA domains, and binds Tfb5/p8 to modulate Ssl2 activity, and anchors Ssl2/XPB to TFIIH [65,66]. Tfb2 is required for stable interactions with Ssl2, Tfb3, Tfb4, and Tfb5, and the HEAT domain plays an unexpected role in association with Rad3, Tfb3, and Tfb4 [67]. In this study, we observed that MoMkt1 interacts with MoTfb2 via BiFC and Co-IP. Furthermore, we found that the MoTfb2 OE affected the pathogenicity of *M. oryzae*. Transcriptome analysis suggested that *MoMKT1* is involved in DNA repair. Based on these results, we speculated that both MoMkt1 and MoTfb2 synergistically participate in DNA damage repair to regulate the growth development and pathogenicity. These results further our understanding of the mechanism of MoMkt1 function in *M. oryzae*.

## Conclusions

**Figure 11.**
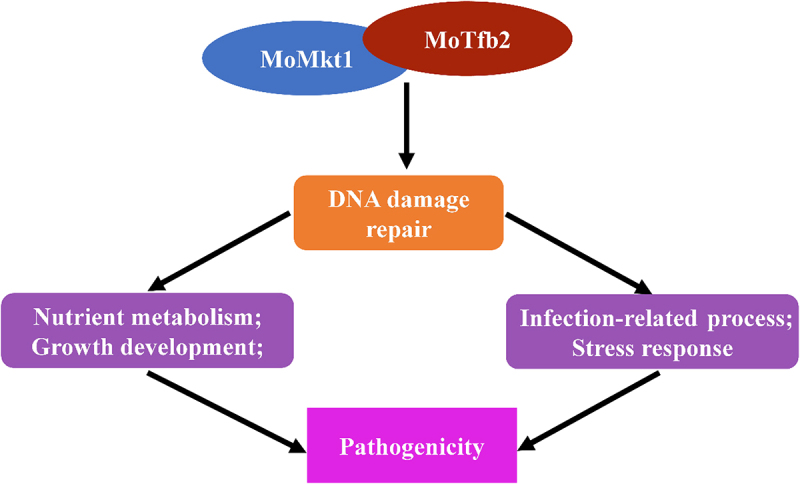
Work model of MoMkt1 in *M. oryzae*. MoMkt1 is an XPG/Rad2 family member that interacts with MoTfb2 to participate in DNA damage repair to regulate growth development and pathogenicity in *M. oryzae*.

In summary, we uncovered the involvement of *MoMKT1* in the growth development, DNA damage response, and pathogenicity of *M. oryzae*. Furthermore, *MoMKT1* regulates metabolic pathways, cell wall, oxidation-reduction processes, and DNA repair processes and interacts with the TFIIH core subunit MoTfb2 (Figure 11). These findings provide insights into MoMkt1-mediated development and pathogenicity in *M. oryzae* and will enrich research on the DNA damage response in plant pathogenic fungi.

## Supplementary Material

Supplementary information.docx

## Data Availability

The data that support the findings of this study in this manuscript and its supplemental materials are available at http://doi.org/10.6084/m9.figshare.27649200.
