# Supplementary material for: MoMkt1, a member of XPG/RAD2 nuclease family, regulates development and pathogenicity in Magnaporthe oryzae
Source: Virulence. 2025 Aug 25;16(1):2546068. doi: 10.1080/21505594.2025.2546068 (PMC12380227; doi:10.1080/21505594.2025.2546068)
Supplement: Supplementary information.docx [file KVIR_A_2546068_SM9480.docx]

**Supplementary information**

**Fig. S1.** Targeted gene deletion of *MoMKT1* in *M. oryzae*. (A) Schematic representation of the targeted deletion of *MoMKT1* using the homologs recombination method. The entire *MoMKT1* gene was replaced with a hygromycin resistance cassette (*HPH*). (B) Detection of knockout transformants (#12, #14, #18, #19, #30, and #34) by PCR with primers P1/P2 and P1/P3. (C) RT-PCR verification of ∆*Momkt1* mutant transformants (#12, #14, #18, #19, #30, and #34) and the complemented transformants (HB: ∆*Momkt1/MoMKT1*). The β-tubulin gene (*MGG_00604*) was used as an endogenous reference. (D) Southern blotting analysis of *Xho* I and *BamH*I-digested genomic DNA from the wild-type strain Guy11 and ∆*Momkt1* mutant transformants (#12, #14, #18, #19, #30, and #34). An approximately 1 kb downstream fragment was used for probe.

**Fig. S2.** The verification of *MoTFB2* overexpression strain *OE-MoTFB2* by RT-PCR. (A) RT-PCR verification of the overexpression strain *OE-MoTFB2*. (B) The β-tubulin gene (*MGG_00604*) was used as an endogenous reference.

**Fig. S3.** MoMkt1 does not interact with MoTfb2 in *vitro*. Y2H assay of the interaction between MoMkt1 and MoTfb2. MoMkt1-AD and MoTfb2-BD were co-transformed into yeast strain Y2H Gold and cultured in SD-Leu-Trp and SD-Ade-His-Leu-Trp medium added with X-α-gal.





**Fig. S1**

**
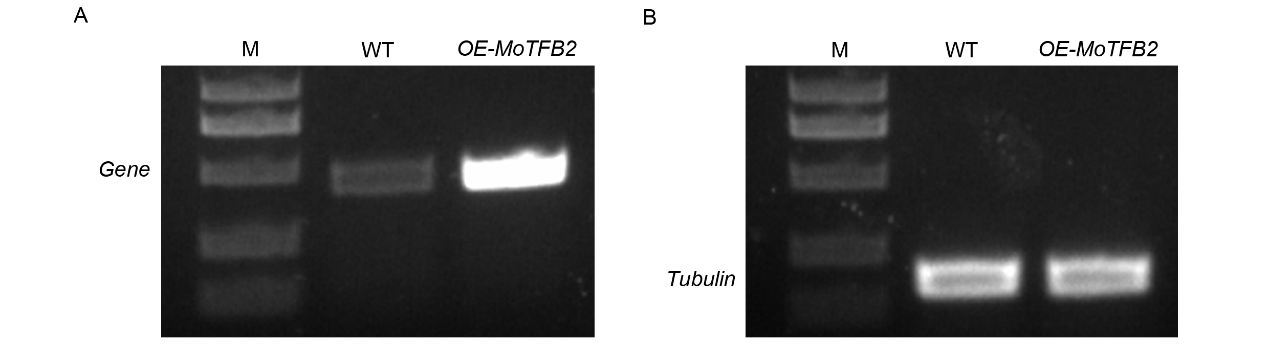
**

**Fig. S2**

**
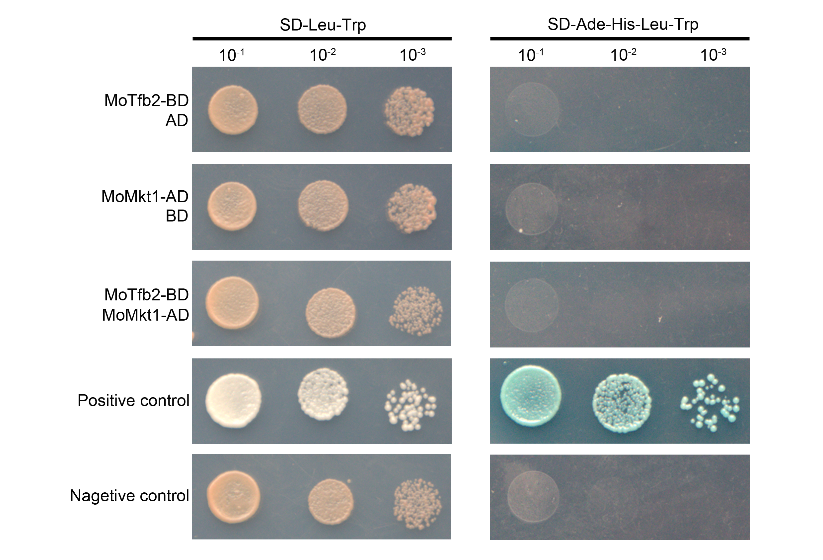
**

**Fig. S3**

**Table S1** Primers used in this study.

| **Primer name** | **Sequences (5’-3’)** | **Remark** |
| --- | --- | --- |
| MoMkt1_upF | GGTACCCGGGGATCCTCTAGAGTGATGAAGGACGTATCGACCAGCA | Amplification of upstream fragment of *MoMKT1* for the gene deletion |
| MoMkt1_upR | TTCATTGTTGACCTCCACTAGTTGGTTCTGCCTAGCTCCCTTCA |  |
| MoMkt1_downF | GCAAAGGAATAGAGTAGATTGCAGCTGGGTACACTCTGTTTGAG | Amplification of downstream fragment of MoMKT1 for the gene deletion |
| MoMkt1_downR | ACGACGGCCAGTGCCAAGCTTGTAATCGCTGCAAACCAGTCAGGA |  |
| MoMkt1_P1 | GTACATCGAGTCCATGAGGCCCT | Amplification of the 5’ upstream fragment using for PCR to confirm the deletion of MoMKT1 |
| MoMkt1_P2 | AAGGCGGAAGGGACACTCACAA |  |
| MoMkt1_P3 | TGCTCACCGCCTGGACGACTAAA |  |
| MoMkt1_P4 | GGGAAACGGCTACCAAGTATCTCGA | Amplification of the 3’ downstream fragment using for PCR to confirm the deletion of MoMKT1 |
| MoMkt1_P5 | GAAGCGCCCGAAGCATAACAAA |  |
| MoMkt1_P6 | AGTACTCGCCGATAGTGGAAACCGAC |  |
| MoMkt1_RT_F | GTCGAGGATGAGTACATCAACGGCCA | Amplification of the fragment using for RT-PCR to confirm the deletion of MoMKT1 |
| MoMkt1_RT_R | CCATGATACCGGCGCATTGGTCT |  |
| β-tubulin_qF | CCAGCCTTCAGTCCTGGGTC | Amplification of the fragment using for RT-PCR and qRT-PCR as an internal parameter |
| β-tubulin_qF | AGGGCAGTGATCTCCTTCTG |  |
| MoMkt1_TZ_F | AGCTGGGTACACTCTGTTTGAGGG | Amplification of the fragment using for southern blot to confirm the deletion of MoMKT1 |
| MoMkt1_TZ_R | CAGGGTCTACACTGTGACAATATCTCG |  |
| MoMkt1_pYF11_F1 | ACTCACTATAGGGCGAATTGGGTACTCAAATTGGTTCTATAGGCACACCCGGAATCTTGCG | Amplification of the native promoter region and full length ORF of MoMKT1 for fusion with GFP tag |
| MoMkt1_pYF11_R1 | GTTGTCGAGCCCATTCATACCACCC |  |
| MoMkt1_pYF11_F2 | AGTACTTGCTGCATACCCCGCC |  |
| MoMkt1_pYF11_R2 | CACCACCCCGGTGAACAGCTCCTCGCCCTTGCTCACCTTTCTTAATTCGAGATACTTGGTAGCCG |  |
| MoTfb2_Flag_F | AACCCGGGCTGCAGGAATTCATGTCTCAGCAACCGGTCATTAC | Amplification of the full length ORF of MoTFB2 for fusion with Flag tag |
| MoTfb2_Flag_R | ATAAGCTTGATATCGAATTCGGCAGCAAGCTTCTTACGGTG |  |
| MoMkt1_YFPN_F | GTCTATATCATGGCCTCTAGAATGCCTTGTGAGTGTCCCTTCC | Amplification of the full length ORF of MoMKT1 for fusion with N-terminal YFP tag |
| MoMkt1_YFPN_R | GTCGCTTACTGCAGGTCGACCTTTCTTAATTCGAGATACTTGGTAGCC |  |
| MoTfb2_YFPC_F | CAATCACAATGGCCGGATCCATGTCTCAGCAACCGGTCATTAC | Amplification of the full length ORF of MoTFB2 for fusion with C-terminal YFP tag |
| MoTfb2_YFPC_R | CTTGCAGGCCGGGCGCCCGGGGGCAGCAAGCTTCTTACGGTG |  |
| MoTfb2_pKD3_F | TCAATCACAATGGCCGGATCCATGTCTCAGCAACCGGTCATTAC | Amplification of the full length ORF of MoTFB2 for fusion with GFP tag |
| MoTfb2_pKD3_R | GCCCTTGCTCACCATCCCGGGGGCAGCAAGCTTCTTACGGTG |  |
| MoTfb2_RT_F | GTCTTCCGATACCACCACAAGTCGA | Amplification of the fragment using for RT-PCR to confirm the overexpression of MoTfb2 |
| MoTfb2_RT_R | CCAAAGTCGTTCAAATCAGGCAGC |  |
| Atf1_qF | CGGCAAACGGCCTCTTTATG | qRT-PCR of ATF1 |
| Atf1_qR | AGGTGACGTCTTGATGGCAG |  |
| Hyr1_qF | ATGGCTTCCGCTACGACAAT | qRT-PCR of HYR1 |
| Hyr1_qR | TTGGAGGCCGTGTTGACTAC |  |
| Trx2_qF | TTTCAAATCCGGTCGCTTGC | qRT-PCR of TRX2 |
| Trx2_qR | TTGTTGCTGATTGCGACGTG |  |
| Ccp1_qF | CCGTGTACAACGACATTGCC | qRT-PCR of CCP1 |
| Ccp1_qR | CTCCTTGTCGTAGGTACCGC |  |
| Nmo1_qF | AGGAGTCAGGCGACAATTCC | qRT-PCR of NMO1 |
| Nmo1_qR | CTCTTCTTTGCCTCGTCCCT |  |
| Lhs1_qF | AACCAGCTCGAGGGCTTTAC | qRT-PCR of LHS1 |
| Lhs1_qR | TTTCTCGAGCTTTGTCCGCT |  |
| Msh1_qF | TGTCTTCACGGCCAGAGCTCAAG | qRT-PCR of MSH1 |
| Msh1_qR | CGCACCACCGAAGTGCTTTTG |  |
| Chk1_qF | AACAATCGGTCGTGGGGCTTATG | qRT-PCR of CHK1 |
| Chk1_qR | GGGATGCTGGCCAATATGTGAATG |  |
| Cds1_qF | GACTACGATGAAGACTCCCAAC | qRT-PCR of CDS1 |
| Cds1_qR | GAAAACCCCGAATTCTGCTTG |  |
| Rad17_qF | AGATGGTATCAGGTTCACGGCCG | qRT-PCR of RAD17 |
| Rad17_qR | TCGTATTCTTCTCCGCCTTGAGCA |  |
| MGG_13429_qF | GGCTACGAATTGCGGGTGACAT | qRT-PCR of 11 DEGS |
| MGG_13429_qR | GCAAGAATCCACGGGTGGCATAT |  |
| MGG_07602_qF | CATTGCTTGGTCGGTGGCTGAT |  |
| MGG_07602_qR | TCCTAGACTTGGACGGCTCGATGA |  |
| MGG_09608_qF | AATCCCACAAGCTCGGAAGGAACT |  |
| MGG_09608_qR | GGATTATGGGGAGCTCAATCCCAG |  |
| MGG_14244_qF | AGGCCCACGAAACAACCATCGT |  |
| MGG_14244_qR | CTGAAACCTGCATGGATCTGGCA |  |
| MGG_02593_qF | AATTGTACGCCGGCAGGGTCAT |  |
| MGG_02593_qR | ATCGGACTTCTCTGACAATCTCGCC |  |
| MGG_04751_qF | TTATGCACCCGGTCAGGAAATCC |  |
| MGG_04751_qR | CTCGTCCTCGAAAACCTCTCCAGA |  |
| MGG_04360_qF | CTCGACCTACGTCTTGCCGTTGA |  |
| MGG_04360_qR | ATCTGAGTGTTGCGAGGGCACTG |  |
| MGG_03329_qF | CGTACATCCTGCACGGAGAATTCC |  |
| MGG_03329_qR | TCCTTGTCGCTCTTCTTCTCACCG |  |
| MGG_13239_qF | ACGGGACTGTGGGAAAGTCTGGAT |  |
| MGG_13239_qR | AGACCATGGGCTTGGCAGACCTA |  |
| MGG_04627_Qf | AGAATGGTTCGGAAAAAGCCGG |  |
| MGG_04627_qR | CTGCTCTCACCACCCATCATTCTG |  |
| MGG_00244_qF | TCCCCGAGTGACGGTATGAATGG |  |
| MGG_00244_qR | CAAACAGGACCTTGTTGCCCGTC |  |
| β-tubulin_qF | CTGCCATCTTCCGTGGAAAGG | Amplification of the fragment using for qRT-PCR of DEGs as an internal parameter |
| β-tubulin_qR | GACGAAGTACGACGAGTTCTTG |  |

**Table S2** List of DEGs (fold change >2; *p* <0.05) in the Δ*Momkt1* mutant (-19) compared to the wild-type strain Guy11 (WT).

**Table S3** Verification of transcriptome analysis by qRT-PCR analysis with randomly selected DEGs.

| **Transcriptome** | | | | **qRT-PCR** | |
| --- | --- | --- | --- | --- | --- |
| Gene | log_2_(fc) | *P* Value | FDR | Fold change | *P* Value |
| *MGG_13429* | -12.346606 | 3.97E-18 | 1.27E-16 | 0.158279435 | 6.32433E-06 |
| *MGG_07602* | -10.0453 | 2.33E-16 | 6.53E-15 | 0.064424656 | 3.94173E-10 |
| *MGG_09608* | -2.94953 | 0.018779 | 0.049742 | 0.714765 | 0.000208 |
| *MGG_14244* | -2.59637 | 1.33E-07 | 1.25E-06 | 0.423553 | 0.000109 |
| *MGG_04751* | -2.09393 | 9.42E-08 | 9.16E-07 | 0.505670448 | 1.44966E-05 |
| *MGG_04360* | -1.57519 | 0.010782 | 0.031063 | 0.901869 | 0.045762 |
| *MGG_04627* | -1.17532 | 0.009346 | 0.027529 | 0.652236 | 0.000325 |
| *MGG_00244* | -1.10954 | 0.008376 | 0.025075 | 0.8553 | 0.000329 |
